# How the gut microbiota impacts neurodegenerative diseases by modulating CNS immune cells

**DOI:** 10.1186/s12974-025-03371-0

**Published:** 2025-03-03

**Authors:** Philipp Schaible, Julia Henschel, Daniel Erny

**Affiliations:** 1 Institute of Neuropathology, Medical Faculty, University of Freiburg, Breisacher Str. 64, 79106 Freiburg, Germany; 2 Faculty of Biology, University of Freiburg, Freiburg im Breisgau, Germany

**Keywords:** Alzheimer’s disease, Microbiota, SCFAs, CNS macrophages, Microglia, CAMs, BAMs, Astrocytes

## Abstract

Alzheimer’s disease (AD) is the most common neurodegenerative disease worldwide. Amyloid-β (Aβ) accumulation and neurofibrillary tangles are two key histological features resulting in progressive and irreversible neuronal loss and cognitive decline. The macrophages of the central nervous system (CNS) belong to the innate immune system and comprise parenchymal microglia and CNS-associated macrophages (CAMs) at the CNS interfaces (leptomeninges, perivascular space and choroid plexus). Microglia and CAMs have received attention as they may play a key role in disease onset and progression e. g., by clearing amyloid beta (Aβ) through phagocytosis. Genome-wide association studies (GWAS) have revealed that human microglia and CAMs express numerous risk genes for AD, further highlighting their potentially critical role in AD pathogenesis. Microglia and CAMs are tightly controlled by environmental factors, such as the host microbiota. Notably, it was further reported that the composition of the gut microbiota differed between AD patients and healthy individuals. Hence, emerging studies have analyzed the impact of gut bacteria in different preclinical mouse models for AD as well as in clinical studies, potentially enabling promising new therapeutic options.

## Introduction

On November 3, 1906, Alois Alzheimer presented a clinical course of a disease, later named after him. More than 100 years later, Alzheimer’s disease (AD) is one of the greatest health care challenges of the twenty-first century worldwide, with more than 55 million patients to date and with forecasts of 150 million patients by 2050 [115]. The clinical symptoms of AD patients include irreversible progressive loss of cognitive function, including memory, spatial orientation, vision and language, ultimately leading to impaired ability to perform activities of daily life [106]. Macroscopically, AD brains exhibit signs of atrophy and enlarged ventricles due to neuronal loss. Histologically, AD is characterized by extracellular amyloid-beta (Aβ) deposition, abnormally phosphorylated tau protein aggregation, synaptic degeneration, and the activation of microglia and astrocytes [102]. Although the pathogenesis is well described, the potential cause(s) of AD remain elusive, hence, preventative or disease-modifying treatments are still limited.

For the past decade, it has been proposed that microorganisms residing in the gut (collectively called microbiota) may be associated with AD. Notably, it has been reported that patients with AD exhibit changes in their microbiota composition and diversity. There is increasing evidence that crosstalk between the gut microbiota and the brain, especially through bacteria-derived mediators such as short-chain fatty acids (SCFAs), plays a critical role in host health, homeostasis and disease [30]. Given the importance of this topic, we review recent findings on how the gut microbiota shapes neurodegenerative diseases by modulating CNS macrophages in mouse models of AD and the potential translation of these findings to therapeutic options for humans.

### Hallmarks of AD: Amyloid-β and phosphorylated tau pathology

The detection of Aβ pathology is a hallmark for AD diagnosis, whereby this 40–42 amino acid peptide is derived from proteolytic cleavage of the amyloid precursor protein (APP). APP is a family of conserved type I membrane proteins that are highly expressed in neuronal and glial cells, with orthologs identified across different species [118]. APP can be processed via α- (ADAM10), β- (BACE1) and γ- (PSEN1/2) secretases into its cleavage products [192]. During pathological amyloidogenic processing by β- and γ-secretases, APP is sequentially cleaved, resulting in C-terminal fragments (CTFs), which are further processed into Aβ40 and Aβ42 fragments [177]. CTF accumulation during APP processing is neurotoxic and can be detected in human cerebrospinal fluid (CSF) [92]. A critical role for Aβ is also evident by the fact that Aβ deposits are now targeted by monoclonal antibodies [81]. Monoclonal antibodies such as aducanumab, lecanemab, and donanemab were generated to target Aβ. Lecanemab, also a humanized IgG1 monoclonal antibody, can bind with high affinity to small soluble Aβ protofibrils, therefore delaying cognitive impairment in the early stage of the disease. This antibody has passed a phase III trial in the USA, although it is still in the approval process in Europe. Currently, one limitation is that the treatment starts at a relatively late stage of the disease and these new therapeutic options are often given after a substantial number of neurons are already lost [172]. Therefore, molecular biomarkers are critical for an early and reliable diagnosis of AD. CSF and blood biomarkers show high potential to optimize diagnostic strategies, with several candidates currently being investigated such as Aβ42/40 as well as p-tau 231 and p-tau217 [6].

In addition to Aβ plaques, tau-mediated neurofibrillary tangles (NFTs) constitute the second main pathological hallmark of AD. The occurrence of NFTs progressively during the course of AD [100], whereby the preclinical phase is characterized predominantly by early Aβ deposition with an onset of clinical symptoms at least 10–20 years later [99]. Compared with Aβ, NFTs are more closely associated with synaptic loss, neurodegeneration, and cognitive decline [121]. Tau is expressed predominantly in neurons and to a lower degree in oligodendrocytes and astrocytes [76]. The main function of tau is to bind to microtubules to stabilize and support their assembly. Furthermore, tau participates in axonal transport [157], axonal elongation, neurogenesis [61], and synaptic plasticity. In AD, tau loses its physiological function with its binding equilibrium for microtubules [7], resulting in an increased cytosolic concentration of unbound tau, causing misfolding, aggregation and the formation of NFTs. NFTs impair regular axonal transport and lead to synaptic dysfunction and neurodegeneration [135].

One percent of all AD cases are familial autosomal dominant, with onset as early as 30 years of age. This familial AD is caused by mutations in APP genes or those associated with APP processing, such as PSEN1 and PSEN2 [95]. However, the etiopathology of the much more frequent sporadic AD remains unclear (reviewed in [139]), probably because the disease pathogenesis is heterogeneous and caused by a complex interaction of genetic and environmental risk factors. Genome-wide association studies (GWAS), revealed specific mutations within risk genes for AD, including the apolipoprotein E (*APOE*4) gene on chromosome 19 [88, 151, 184]. *APOE* has critical functions in homeostasis, with APOE4 carriers showing accelerated breakdown of the blood–brain barrier (BBB) [9, 113]. Additionally, several risk genes, such as *TREM2*, *ABCA7*, *CD33* and *MS4A6A, are expressed by CNS macrophages* [88, 151, 183]. In the human brain, *TREM2*, which encodes an immunoreceptor tyrosine-based activation motif-containing cell surface receptor, is among the most highly expressed receptors on microglia [28]. Heterogeneous mutations within *TREM2* increase the risk of late-onset AD by 2–fourfold [52, 154], suggesting a potentially critical role for microglia during AD already from disease onset on [102].

### The role of microglia in AD

Microglia are the resident macrophages of the CNS with a plethora of immune and homeostatic functions, including innate immune response, neuroprotection, synaptic pruning and phagocytosis of cellular debris [124]. In 1919, Río-Hortega published a series of papers, where he first described `microglia` as an independent cell type and defined their distribution and morphological phenotype. Interestingly, he also noted the putative mesodermal origin of these cells and recognized their phagocytic capacity [33, 152]. In 1999, it was proposed that microglia originate from the yolk sac [122], which was later confirmed by two fate-mapping studies [48, 144]. Moreover, microglia are derived from CD45^−^ c-kit^+^ erythromyeloid progenitors in the yolk sac, and their developmental process is characterized by the maturation and differentiation of microglia progenitors via CX3CR1^−^ and CX3CR1^+^ stages (Kierdorf et al. 2013). Microglia are highly plastic and regulate the stability of their microenvironment in the healthy CNS independently of hematopoietic stem cell (HSC)-derived cells [57]. They are long-living cells with low turnover rates across different brain regions, however they can locally proliferate during perturbation [164].

With the help of *Cx3cr1*^*GFP/WT*^ reporter mice [72], it was demonstrated that microglia are constantly active, probing an area tenfold larger than their cell body [32, 116]. To recognize microbes, metabolites, chemokines and cytokines, microglia express a variety of pattern recognition receptors (PRRs), such as toll-like receptors (TLRs), NLRP3 and scavenger receptors, including the Aβ-sensing CD36 [102]. Upon Aβ recognition, microglia accumulate around Aβ plaques and clear Aβ deposits, e.g., through phagocytosis. Concomitantly, these plaque-associated microglia are in an activated state and initiate proinflammatory signaling cascades as well as inflammasome formation [179]. During disease progression, microglial morphology changes as they become less ramified, with larger cell bodies and shorter processes and a decrease in their process dynamics [85]. During disease progression, microglia continuously produce neurotoxic cytokines such as interleukin (IL)-1β, IL-8, tumor necrosis factor alpha (TNF-α) and reactive oxygen species (ROS) [54]. However, they lose their ability to efficiently clear Aβ over time [84]. This chronic immune activation can lead to an exhausted microglial state and downregulation of Aβ-degrading enzymes such as insulin-degrading enzyme (IDE) and neprilysin (NEP) (Wyss-Coray et al. 2002). Furthermore, homeostatic genes, such as *P2ry12* or *Tmem119,* are downregulated upon microglial activation, whereas *Apoe, Clec7a* and *Trem2* are upregulated in plaque-associated microglia [78]. Specifically, *TREM2* upregulation has been found both in patients with AD and additionally in transgenic mouse models [71] [78] (**Box 1**). A potentially TREM2-dependent neuroprotective role of plaque-associated microglia in the early stages of plaque formation was reported in mouse models as well as human AD patients and may attenuate tau seeding in neuritic plaques [75, 96, 189].

In addition to beneficial roles of Aβ recognition and clearing, microglia may also enable the spread of Aβ [34], and in addition the tau protein between neurons via phagocytosis and exocytosis [5, 163], although the exact mechanisms are not yet fully understood. A study with human and murine samples suggested that microglia can take up tau but do not completely degrade it, consequently triggering tau aggregation in recipient cells [62]. Furthermore, a study using a mouse model for tauopathy described microglial NF-κB activation and the resulting release of cytokines, such as IL-1, IL-6 and TNF-α, as a possible cause for microglial-mediated tau spreading [175].

### CNS-associated macrophages

CNS-associated macrophages (CAMs), also known as border-associated macrophages (BAMs), have become increasingly important in recent years. CAMs are present at the interfaces between the periphery and brain parenchyma, including the leptomeninges (harboring leptomeningeal macrophages, mΜΦ), perivascular space (containing perivascular MΦ, pvΜΦ) and choroid plexus (comprising stromal choroid plexus MΦ, cpΜΦ and Kolmer epiplexus cells), controlling these potential gateways into the CNS [129]. CAMs and microglia share a common progenitor during embryonic development, and the final cell fate occurs subsequently and locally in the developing CNS [49]. Interestingly, leptomeninges function as an intermediate environmental niche during early postnatal development, from where mΜΦ migrate into the perivascular space to give rise to pvMΦs [104]. In contrast, cpΜΦs are an ontogenically and transcriptionally mixed population partially replenished with HSC-derived cells in adulthood [104]. In contrast to microglia, the development of CAMs has recently been shown to be independent of transforming growth factor β (TGF-β) [19, 171]. In the context of AD, pvΜΦs seem to play a role in clearing Aβ across the BBB [58, 161]. Notably, several risk genes for AD are also strongly expressed in human pvΜΦ [183]. In a slow-progressing *App*^NL−F^ AD mouse model, secreted phosphoprotein 1 (SPP1/osteopontin) was upregulated predominantly in pvΜΦs and proposed to be required for microglia to engulf synapses and enhance phagocytosis in the presence of Aβ oligomers [143].

### Astrocytes and AD

In addition to microglia and CAMs, astrocytes feature certain immune regulatory functions. These distinct glial cells with a neuroectodermal origin, firstly termed astrocytes 1895 by Michael von Lenhossék, are the most abundant cells within the CNS [46]. Astrocytes maintain plenty of essential homeostatic functions during development and adulthood, especially the control of the BBB (reviewed in great detail in [91]). Aβ and tau accumulation lead to astrocytic activation [147] and the upregulation of glial fibrillary acidic protein (GFAP) [40]. Elevated GFAP levels can be measured in the blood and CSF of AD patients, whereby plasma GFAP is suggested to increase early in AD progression [10]. Activated astrocytes undergo functional and molecular changes [43] and contribute to neurodegeneration via the release of neurotoxic cytokines, nitric oxide (NO) and ROS [15]. Interestingly, astrocytes also express major AD risk genes, including *APOE*, *CLU* and *FERMT2* [4], further supporting a pivotal role for glial cells in AD.

### The gut microbiota steers CNS immunity

Increasing evidence suggests that environmental factors, including the intestinal microbiota, critically shape the host’s physiology and are essential for immune function, including the CNS [42]. The word microbiota is composed of ancient greek μικρός *(mikrós)* 'small' and βίος *(bíos)* 'life' and includes bacteria, archaea, viruses, fungi and protozoa that reside in and on humans, animals and plants. Approximately 2500 years ago, the ancient Greek physician Hippocrates proclaimed `all disease begins in the gut`. Two millennia later, the development of new techniques boosted microbiological research, underscoring the importance of the gut microbiota in health and disease. Today it is known, that the gut-brain axis enables bidirectional communication between the CNS and the gut. This communication occurs through direct and indirect neuronal connections and endocrine, metabolic and immune pathways via vitamins, neurotransmitters, and microbial metabolites such as SCFAs [21]. Increasing evidence suggests that SCFAs, including acetate, propionate and butyrate, produced predominantly by gut bacteria through the fermentation of otherwise indigestible dietary fibers are important gut-derived mediators able to substantially shape immune function in general [162]. Accordingly, SCFAs have also been associated with a variety of (neurodegenerative) diseases [30]. Specifically, acetate was identified as the essential SCFA driving microglia maturation and regulating their homeostatic metabolic state. Acetate normalized the immature phenotype of microglia that was present in germ free (GF) mice as well as an impairment of complex II in the mitochondrial respiratory chain [41].

**Fig. 1:**
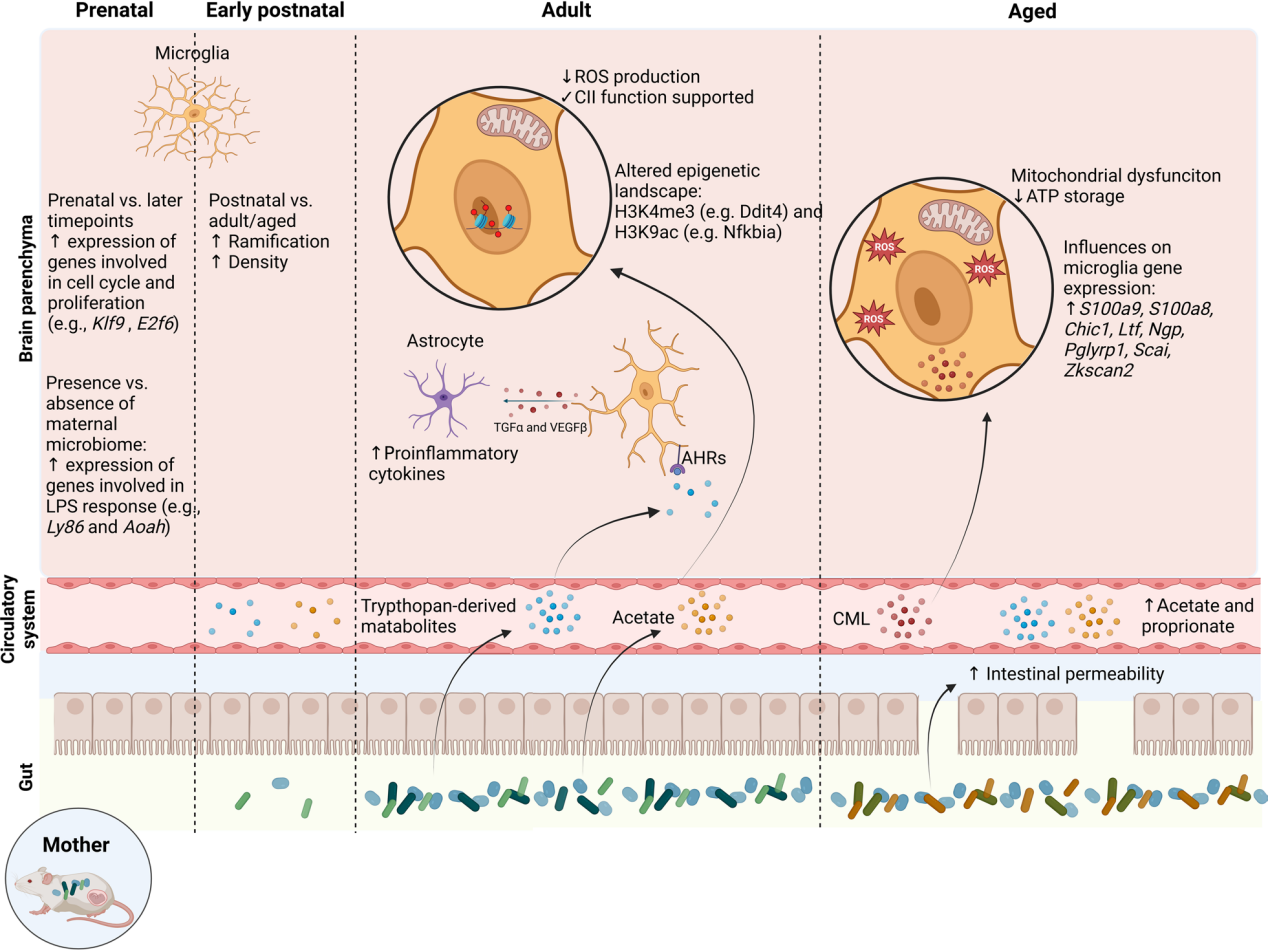
Gut-derived molecules affect the CNS immune system during development and aging. Before birth the microbiota of the mother affects the microglia gene expression of the embryo, e.g. by increasing genes associated with LPS stimulation. After birth the pub is colonized and bacterial amount and diversity increases. During adulthood a stabile microbiota composition is reached, which modulates microglia and astrocytes via microbial metabolites, including the SCFA acetate and tryptophan-derived metabolites. In aged mice *N*^6^-carboxymethyllysine (CML) accumulates in the brain, which results in oxidative stress and mitochondrial dysfunction in microglia

Already during embryonic and early postnatal development, the maternal microbiota significantly influences microglial properties in a time- and sex-dependent manner (Fig. 1) [168]. Later in life, the human and mouse gut microbiota display an age-specific composition [132]. Furthermore, intestinal permeability is partially increased, resulting in higher blood concentrations of gut-derived metabolites. It was shown that gut-derived *N*^6^-carboxymethyllysine (CML) augmented oxidative stress and mitochondrial dysfunction in microglia during aging [114]. Moreover, gut-derived tryptophan metabolites can directly act on microglia via aryl hydrocarbon receptors (AhR) [133]. Microglial AHR activation further regulates astrocytes via transforming growth factor α (TGF-α) and vascular endothelial growth factor B (VEGF-B). In summary, gut-derived molecules, including tryptophan, CML, and SCFAs, can affect CNS immunity during steady state and disease.

Box 1: Mouse models for Alzheimer´s Disease To investigate the pathology of AD, sufficient mouse models are needed. Since wild-type mice don´t naturally develop Aβ plaques and neurofibrillary tangles, transgenic mouse models were developed. Among others, the most commonly used models for Aβ pathology are APPPS1, APPPS1-21 and 5xFAD transgenic mice.

**APPPS1 (APPSWE/PS1ΔE9)**

APPPS1 mice express humanized APP harboring the Swedish mutation (K670N/M671L) and human PSEN1 missing exon 9 [69]. Aβ accumulation and astrogliosis can be observed from 6 months on with prominent Aβ accumulation in the hippocampus and cortex at 9 months [69]. Mild neuronal loss can be observed near plaques, beginning at 8 months [67], whereas neurofibrillary tangles don´t occur in APPPS1 mice. Cognitive impairments can be observed, including decreased memory function [80], spatial learning [87] and nest-building behavior [70].

**APPPS1-21**

APPPS1-21 mice contain the APP Swedish mutation (K670N/M671L) and mutated human PSEN1 (L166P) transgenes under direction of a neuron-specific *Thy1* promoter. Compared to APPPS1 mice, APPPS1-21 show a more aggressive phenotype with Aβ accumulation beginning at 2 months in the neocortex and at 4 months in the hippocampus. This model shows neuronal cell loss in the dentate gyrus at 17 months and impaired spatial memory at 8 months [131].

**5xFAD**

5xFAD mice express human APP with 3 mutations found in familial AD (Swedish (K670N/M671L), Florida (I716V), and London (V717I)) and two mutations in PSEN1 (M146L and L286V) [119]. The transgenes are expressed under the Thy1 promoter and produce APP levels up to three times that of endogenous APP [120, 136]. This aggressive mouse model causes Aβ plaque formation, astrogliosis and microgliosis at 2 months, starting in the subiculum and layer V of the cortex [119]. At 6 months, Aβ accumulation can be found in various brain regions, including the olfactory bulb, brainstem and thalamus. Furthermore, at 6 months neuronal loss can be seen in brain regions with intense Aβ pathology [119]. This pathology leads to behavioral impairment with reduced spatial working memory beginning at 4 months [119] and altered social behavior beginning at 9 months [45].

**APPNL^−G−F^**

The APP^NL−G−F^ mouse model features the knock-in of the Swedish (NL), Arctic (G) and Liberian (F) mutation into APP [138]. Pathogenic Aβ production and aggregation is increased, while APP is expressed at wild-type levels, leading to AD-associated pathologies, including synaptic loss, microgliosis and astrocytosis. APP^NL−G−F^ mice display Aβ plaques from 2 months of age (reaching a plateau at 7 months) [138], synaptic degeneration begins at 3–4 months of age [89] and subtle memory impairments at 6 months [90]. However, APP^NL−G−F^ mice don´t develop neurofibrillary tangles and neurodegeneration is not detectable [138].

**PDAPP (line109)**

This line is one of the first mouse models of AD and was initially described in 1995. This line displays high human APP expression with multiple isoforms [47]. The deposits of human Aβ starts at 6–9 months of age in the hippocampus, corpus callosum, and cerebral cortex, also tau immunoreactivity is detected after 14 months [103]. Cognitive deficits can be seen from an age of approximately three months. [35].

**P301S (line PS19)**

This mouse line expresses mutant human microtubule-associated protein tau (MAPT) driven by the Prnp promoter [50]. Mice show neuronal cell loss and brain atrophy by 8 months, starting in the hippocampus [187]. Behaviorally, the mice display signs of age-associated cognitive impairment starting at 6 moths, including selective deficits in spatial learning and memory ability.

## Modulation of the gut microbiota shapes disease hallmarks of AD

### Induced microbiota modulation by antibiotics results in diminished Aβ accumulation

Initially, Minter and colleagues uncovered that alterations in the gut microbiota affect Aβ pathology in the CNS [111]. They used the APP/PS1 mouse model (Box 1) and treated them with a cocktail of several antibiotics (ABX) by oral gavage, starting at postnatal day 14 (P14) until P21, followed by ABX supplementation in the drinking water until the age of 6 months (Table 1) [111]. Compared with vehicle-treated APP/PS1-21 mice, long-term ABX-treated male APP/PS1 mice showed a significantly decreased Aβ burden with smaller plaques in the hippocampus and cortex. In follow-up studies, they confirmed the influence of oral ABX treatment on Aβ deposits in the same mouse model [36, 37]. Interestingly, they reported that only a broad cocktail of ABX (composed of kanamycin, gentamicin, colistin, metronidazole and vancomycin) ameliorated the Aβ burden, whereas the application of the individual substances alone had no effect [36]. These results may indicate that a rather broad reduction of several bacterial families is necessary to affect Aβ accumulation.

**Table 1:** Studies on different AD mouse models reporting the impact of the gut microbiome on the AD pathology

Mouse Model	Gender		Experimental Setup	Microbiome	Disease hallmarks	Glial cells	Additional findings	Reference
APP/PS1- 21	**♀ **and **♂**		• Analysis 14 months old mice: Sodium butyrate (1.2 g/kg) injection for 6 weeks • Analysis: 15 months		• Associative memory formation ↓ in APP/PS1-21 compare to WT • SB administration rescue impairment in associative memory without affecting hippocampal and cortical Aβ plaque burden		• Hippocampal and cortical acetylation: H3K9, H3K14, H4K5, H4K8, H4K12, and H4K16 ↓ in APP/PS1- 21 compared to WT • Hippocampus: *Myst4*, *Fmn2*, *Marcksl1*, *Gsk3*, *GluR1*, *Snap25, Prkca, and Shank3 *↑ in SB treated animals	[51]
					♂ Aβ plaque burden ↓ and soluble Aβ ↑ in ABX-treated vs. untreated			
APP/PS1	**♀ **and **♂**		• ABX-mix gavage from p14-p21: gentamicin (1 mg/ml) vancomycin (0.5 mg/ml) metronidazole (2 mg/ml) neomycin (0.5 mg/ml) ampicillin (1 mg/ml) kanamycin (3 mg/ml) colistin (6000 U/ml) cefaperazone (1 mg/ml) • 1/50th ABX-mix in drinking water for the duration of lifespan **→ long-term ABX- treatment** • Analysis: 5 months ♀ and 6 months ♂	Long-term ABX treatment: • α-diversity ↓ • *Akkermansia *and *Lachnospiraceae* ↑ • Bacterial abundance ↔ • Enlarged cecum	♀ Aβ plaque burden ↔	ABX-treated ♂ vs. untreated: • Plaque associated microglia and astrocytes ↓ • Altered microglia morphology: dendrite length ↓ dendrite number and terminal end points ↑	**♂** • Chemokines and cytokines in serum: CCL11, CXCL16, LIX, TIMP-1 and TNFα ↑ in ABX-treated mice compared to untreated	[111]
APP/PS1	**♂**		• ABX-mix gavage from p14-p21: gentamicin (1 mg/ml) vancomycin (0.5 mg/ml) metronidazole (2 mg/ml) neomycin (0.5 mg/ml) ampicillin (1 mg/ml) kanamycin (3 mg/ml) colistin (6000 U/ml) cefaperazone (1 mg/ml) **→ short-term ABX- treatment** • Analysis: 6.5 months	Short-term ABX- treatment: • Bacterial abundance ↔ • *Lachnospiraceae* ↑ • *Akkermansia *and *S24-7 *↓ • Morphological alterations ↔	• Aβ plaque burden and plaque size ↓ • Alteration in APP proteolysis ↔ • Foxp3+ T-regs transcription factor expression levels ↑ in blood and brain ABX-treated compared to untreated	• Plaque associated microglia and astrocytes ↓ but relative to Aβ plaque area ↔ • Altered microglia morphology in ABX-treated mice:dendrite length, dendritenumber and terminal end points ↑	Chemokines and cytokines of ABX- treated vs. untreated in serum: CCL11, IL-1β, IL-2, IL-3 and SCF ↑ and IL-6 ↓ in CSF: IL-2, IL-3 and SCF ↓ and CCL11, IL-1β and IL-6 ↔	[110]
APP/PS1	**♀ **and **♂**		• SPF housing of APP/PS1 and WT littermates • Analysis: 1, 3.5 and 8 months	Age-related shift in SPF APP/PS1 vs. SPF WT at 8 months: • *Firmicutes*, *Verrucomicrobia*, *Proteobacteria Actinobacteria Akkermansia *↓ • *Bacteroidetes Tenericutes S24-7 *↑ • α-diversity ↑				[55]
			• GF housing vs. SPF controls • Analysis: 1, 3. 5 and 8 months		• Hippocampal and cortical Aβ plaque burden ↓ and Aβ- degrading enzymes (NPE and IDE) ↑ in 3.5 and 8 months old GF mice vs. SPF controls	• Microglia density ↓in 3.5 and 8 months old GF mice vs. SPF controls	Cytokines in brain homogenate: • IL-1β ↓in 8 months old GF mice vs. SPF controls • IFN-γ, IL-2 and IL- 5↓ in 3.5 months old GF vs. SPF mice	
			• GF APP/PS1 mice were colonized at 4 months via oral gavaged with cecal	• Akkermansia and Rikenellaceae ↓ and S-24-7 ↑ in	• Aβ38, Aβ40 and Aβ42 ↑ in gavaged GF mice			
			content of 12 months-old SPF WT or SPF APP/PS1 mice • Analysis: 6 months	GF mice gavaged with cecal content of APPPS1 mice vs. WT ceacal content	with a more pronounced effect in mice gavaged with cecal content of APPPS1 mice • Aβ42/ Aβ40 ratio ↔			
APP/PS1- 21	**♀ **and **♂**		• ABX-mix gavage from p14-p21: kanamycin (4 mg/ml) gentamicin (0.35 mg/ml) colistin (8500 U/ml) metronidazole (2.15 mg/ml) vancomycin (45 mg/ml) • 1/50th ABX-mix in drinking water for the duration of lifespan **→ long-term ABX- treatment** • Analysis: 7 weeks and 3 months	Long-term ABX treatment: • microbial diversity after ABX treatment ↓ • ABX-treated female and male ↔ ♂ • Similar α-diversity in gavaged and donor mice	♀ Aβ plaque burden ↔	♀ • Plaque associated microglia number ↔ • Altered microglia morphology in ABX-treated mice • Expression of homeostatic microglia genes: *Mef2a*, *Junb*, *Bhlhe41*, *Fos*, and *Tnfrsf11a *↑ and: *Lgals3*, *C1qa*, *C1qb*, *Cd63*, and *Lag3 *↓ in ABX-treated males only (as determined via RNAseq of the cortex)	♀ Pro-inflammatory metabolic pathways, cytokines and chemokines ↑ in ABX- treated females compared to ABX- treated males	[37]
	♂				♂ • Aβ plaque burden ↓ in ABX treated mice compared to		♂ anti-inflammatory ↑ and pro-inflammatory cytokines and	
			♂ • Daily gastric gavage of fecal content of age- matched APP/PS1-21 mice in ABX-treated APPPS1-21 mice starting at P25 • Analysis: 7 weeks	• α-diversity ↓ in non-gavaged ABX-treated mice • *Bacteroides*, *Prevotella *and *S24-7 *↑ in gavaged mice	• cortical Aβ burden and plaque size ↑ in gavaged mice	• Microglial cell body ↑ and dendritic branch lengths and dendritic branch points ↓ in gavaged mice	chemokines ↓in the plasma of ABX- treated males compared to vehicle- treated males	
5xFAD		**♀ **and **♂**	• SPF housing • Analysis: 2, 3, 5, 7 and 9 months	• Constant shift in gut microbiome composition of 5xFAD mice: *Firmicutes *↑ *Bacteroidetes *and *Verrucomicrob*ia ↓ compared to a constant microbiome of WT mice		Whole brain homogenate: • Two phases of microglia activation in 5xFAD mice at 3 months and starting at 7 months with a progressive ↑ of CD86+ • CD45high cells ↑ in the brain of 5xFAD mice compared to WT mice		[179]
			• WT co-housed with 5xFAD, WT bred and housed separately • ABX-mix gavage for 5 months: ampicillin (0.1 mg/mL) streptomycin (0.5 mg/mL) colistin (0.1 mg/mL) **→ long-term ABX treatment** • Analysis: 7 months • 5xFAD mice orally gavaged for one month with 100 mpk GV-971 starting at 6 months, un- treated 5xFAD mice as controls • Analysis 7 months	• Microbial abundance ↓ in the gut after ABX- treatment • Comparable gut microbiome of 5xFAD and co- housed mice, distinct from the composition of separately housed WT mice • GV-971 alters the microbiome composition in 5xFAD mice	Aβ and tau accumulation ↓ in the hippocampus of 5xFAD mice treated with GV-971 compared to untreated 5xFAD mice Aβ and tau accumulation ↓ in the hippocampus of 5xFAD mice treated with GV-971 compared to untreated 5xFAD mice	Whole brain homogenate: • CD86+ microglia and CD45highCD4+CXCR3+ Th1 cells ↓ in 5xFAD mice after ABX-treatment compared to non-treated 5xFAD mice • Infiltration of Th1 cells ↓ in separately housed WT mice compared to 5xFAD and co- housed WT mice ________________________ Microglia density in the hippocampus ↓of GV-971 treated 5xFAD mice compared to untreated 5xFAD mice and Th1 cells ↓ in whole-brain homogenate	• Discrimination learning ↓ in 5xFAD and co-housed WT animals compared to separately housed WT animals • Improved cognitive performance in GV- 971 treated 5xFAD mice compared to untreated 5xFAD mice Feces and blood: Significant changes in histidine, phenylalanine and isoleucine levels in 5xFAD mice compared to WT mice	
			• Gavage of above- mentioned individual ABX respectively Time of analysis: 9 weeks	• Most pronounced • ↑ in cecal weight after ABX-cocktail and gentamicin treatment alone				
5xFAD		**♂**	• SPF- GF housing Supplementation with ABX- mix containing drinking water for 2 months starting 2 months before time of analysis: vancomycin (1 mg/ml) cefoxitin (1 mg/ml) gentamicin (1 mg/ml) metronidazol (1 mg/ml) • Analysis: 4 and 10 months (microbiome only at 4 months)	• α-diversity and bacterial abundance ↓ after ABX-treatment • SPF 5xFAD mice and non treatet littermates ↔	• Hippocampal Aβ burden and (in)soluble Aβ42 and Aβ40 fraction ↓ in GF and ABX- treated mice • Plaque size in GF mice (at 4 months) and GF and ABX-treated mice (at 10 months) ↓ compared to SPF mice • APP processing ↔	4 months: • Microglia density in the hippocampus ↑ of GF mice compared to SPF mice (after ABX-treatment ↔) • Plaque associated microglia and Aβ uptake ↑ in GF mice compared to SPF and ABX- treated mice • Expression of genes implicated in Aβ detection/clearance ↑ (Apoe, Trem2, Clec7a) and phagocytosis/phagosome maturation. Neuroinflammation signalling pathways ↓ in FACS-isolated hippocampal microglia from GF 5xFAD mice. Interferon signalling ↑ in ABX-treated 5xFAD mice. 10 months: • Microglia density, plaque associated microglia and Aβ uptake ↔ in GF, SPF and ABX-treated mice	• More preserved cognitive performance (T- maze and novel object recognition) of GF and ABX- treated 5xFAD mice compared to SPF 5xFAD mice at 10 months (SPF, GF and ABX-treated WT ↔ animals) • Exclusive neuronal cell loss in the subiculum and CA1 region of SPF 5xFAD mice compared to GF and ABX-treated 5xFAD mice	[109]
APP/PS1		**♀ **and **♂**	• SPF- GF housing • Analysis: 3 months (microbiome) and 5 months (histological hallmarks and behaviour)	• Plasma concentration of the SCFAs acetate, butyrate, and propionate ↑ in SPF mice • compared to GF mice	• Aβ plaque load ↓ in GF mice compared to SPF mice (Number of small plaques ↓ larger plaques ↔)		• Cognitive performance ↑ (Barnes maze) of GF mice	
			• Supplementation of GF APP/PS1 mice with acetate (65.7 mM) butyrate (40 mM) and propionate (25.9 mM) in the drinking water for 2 months starting at 4 weeks **→ short-term SCFAs treatment** • Analysis: 3 months	• SCFA concentration was normalized by the supplementation	• Cerebral Aβ plaque load after SCFA supplementation ↑ compared to GF mice • Aβ plaque load ↑ in SPF mice supplemented with SCFAs compared to SPF mice • No significant differences in APP processing	• Activated morphology and plaque associated microglia ↑ in SCFA supplemented GF mice compared to control-treated mice • Genes associated with microglia activation ↑ ApoE protein expression ↑ in SCFA- compared to control-supplemented GF group		[27]
5xFAD		**♂**	• SPF- GF housing • GF housing: acetate (150 mM) supplementation for 8 weeks via the drinking water • Analysis: 4 months		• hippocampal Aβ ↑ accumulation in GF + acetate and SPF mice compared to GF mice	• Plaque-associated microglia ↓ in GF + acetate and SPF mice compared to GF mice • Ramified morphology of plaque associated microglia ↑ with more mitochondria in GF mice compared to GF + acetate and SPF mice • Metabolic activity and expression of proinflammatory genes ↑ in SPF mice compared to GF mice	Acetate uptake of cultured primary microglia in the presence of Aβ and acetate were determined by exposing them for 6 h with monomeric Aβ (5 mM 13C-acetate and 2 µM Aβ) • Aβ uptake ↓ of microglia in Aβ + acetate cultures compared to Aβ • cultures	[41]
						• Glycolysis, gluconeogenesis and glucose uptake ↓ in GF mice compared to GF + acetate and SPF mice • Aβ uptake ↓ in GF + acetate and SPF mice compared to GF mice	• Cxcl10, Ccl5, and Relb expression ↑ in Aβ + acetate cultures compared to Aβ cultures Acetate uptake and incorporation in TCA cycle intermediates ↑ in Aβ + acetate cultures compared to Aβ cultures	
5xFAD		**♂**	• SPF- GF housing Supplementation with ABX- mix containing drinking water for 2 months starting at 2 months: vancomycin (1 mg/ml) cefoxitin (1 mg/ml) gentamicin (1 mg/ml) metronidazole (1 mg/ml) • Analysis: 4 months		• Hippocampal Aβ accumulation in GF and ABX- treated 5xFAD mice ↓ compared to SPF mice	• mMφ, cpMφ and pvMφ cell density ↑ in SPF 5xFAD mice compared to GF and ABX-treated 5xFAd mice and Wt mice • Aβ uptake ↑ only from pvMφ of GF and ABX- treated 5xFAD mice • compared to SPF 5xFAD mice		[141]
P301S		**♀ **and **♂**	• GF- conventionally housing and colonization • ABX-mix gavage from P16- P22 with 100 µl ABX-mix: kanamycin (4 mg/ml) gentamicin (0.35 mg/ml) colistin (8500 U/ml) metronidazole (2.15 mg/ml) vancomycin (0.45 mg/ml) • Analysis: 10 months	**♂** Difference in microbiome composition with an • ↓ of *Heliobacter*, *Ruminococcus *and *Butyricicoccus *in ABX-treated mice	**♀** • Brain atrophy ↔	Astrocytes and microglia ↓ in GF mice **♂** • GF TE4 mice and ABX- treated TE3 mice showed significantly ↓ phosphorylated tau in the hippocampus compared with their controls	• TE3 (ApoE3) ABX- treated mice: ↓ NK, pDC and altered lung alveolar macrophages gene expression • GF mice: ↓ pDC and γδ tcells	[146]
					**♂** Brain atrophy ↓ in GF TE4 (ApoE4) and ABX-treated mice compared to conventionally • housing and H2O treated mice			
APP/PS1		**♀ **and **♂**	• Supplementation with acetate (67.5 mM) butyrate (40 mM) and propionate (25 mM) in the drinking water for 9 months starting at 5 months old mice **→ long-term SCFAs treatment** • Behavioral tests on 14- months old mice mice	After 2 months SCFA diet: ↑ *Akkermansia *and *Clostridium XIVb* After 9 months • ↑*Anaerofustis*	Aβ and tau phosphorylation↓ significantly reduced in the SCFA supplementations group compared to non-treated APP/PS1 mice	• SCFA supplementation regulated neurotransmitter uptake: ↑Glul, Slc1a2, Gstm1, genes that involved in astrocyte-neuron metabolic coupling • ↑ transcription level of glutamine synthetase • No pro-inflammatory response of microglia		[159]
APP/PS1- 21		**♀ **and **♂**	• SPF- GF housing • ABX-mix gavage from P14- P21 with 200 µl ABX-mix: Kanamycin (4 mg/ml) Gentamicin (0.35 mg/ml) Colistin (8,500 U/ml) Metronidazole (2.15 mg/ml) Vancomycin (0.45 mg/ml) • Time of analysis: 9 weeks • Microglia depletion via PLX5622 treatment from P24 till end of experiment (1200 ppm) in ABX- treated mice (P14-P21) • Analysis: 3 months • Daily FMT from non ABX- treated into ABX-treated (P14-P21) males, starting at P24 till 9 weeks via oral gavage	ABX-treated ♂ • Positive correlation of GFAP+ astrocyte density and *Odoribacter *and *Anaeroplasma* • Positive correlation of plaque associated astrocytes and *Anaeroplasma *and *Paraprevotella* • Negative correlation of astrocyte process number/length and *Odoribacter*, *Muribaculum*, *Paraprevotella*, *Intestinimonas*,	♂ Cortical Aβ ↓load in GF, compared to SPF controls	♂ • GFAP+ (plaque associated) astrocytes ↓ after ABX- treatment, compared to vehicle-treated control • Plaque associated astrocytes in GF ↓ compared to SPF controls • Altered astrocyte morphology in GF and ABX- treated males, compared to SPF (process lengths, areas, number of processes, branch points and terminal points ↑) • GFAP+ (plaque associated) astrocytes ↓ after ABX and PLX5622 treatment, compared to PLX-treated and ABX untreated controls • Morphological differences in PLX treated mice with and without ABX-treatment ↔		[24]
				*Tyzzerella *and *Anaero plasma*		• FMT treatment normalizes astrocyte morphology: process lengths, areas, volume, cell body size, number of processes, branch points and terminal points ↓ compared with ABX-treated male mice		
APPNL-G-F		**♀ **and **♂**	• SPF- GF housing • SPF **♀ **Supplementation with ABX- mix containing drinking water for 2 weeks: Ciprofloxacin (0.2 mg/ml) Ampicillin (1 mg/ml) Metronidazole (1 mg/ml) Vancomycin (0.5 mg/ml) **→ short-term ABX treatment** • Recolonization: GF and ABX-treated mice received FMT from the SPF mice via 2 gavages at d0 and d2 • Gavaged for 3 days with butyrate and propionate (1g/kg body weight) • **→ short-term SCFAs treatment**	levels of propionate and butyrate ↓ in the feces of AppNL-G-F mice compared to the WT	• Propionate and butyrate treatments in AppNL-G-F resulted in a significant ↓ of Aβ accumulation Aβ plaque area and number of plaques ↓	• Propionate and butyrate treatments in AppNL-G-F: significantly ↑ number of microglia in the olfactory bulb • only propionate treatment induced ↑ in the number of microglia in the hippocampus and cerebellum • non- Aβ plaque-associated microglia showed activated microglial Phenotype • GFAP+ astrocyte morphology ↔	Propionate and butyrate-treated AppNL-G-F mice showed significantly ↑ tight junctions expression compared to non-treated AppNL- • G-F	Junhua Xie et al., 2023
5xFAD		**♂**	• 5-week-old mice: microbial strains ( 1 × 109 CFU) or vehicle was maintained for 16 weeks via • oral gavage		• Soluble Aβ42 ↓ in cerebral cortex of mice treated with *Lactobacillus reuteri,*	• Cerebral cortex: Iba1+ ↑ • Hippocampus: Iba1+ ↔	Pro-inflammatory cytokines such as IL1α, IL1β, IL2, IL12, IL17, IFN-γ, and TNFα ↓	[82]
			• Metabolite mixture-with tryptophan and indole-3- lactic acid (0.05 mg/g) for 16 weeks via oral gavage • Analysis: 21 weeks		• *Lactobacillus delbrueckii *and *Streptococcus thermophilus *compared to 5xFAD and mice treated with metabolite mixture	• GFAP+ astrocytes colocalization with Aβ ↑ • in the treatment groups and 5xFAD control group compared to the WT	• IL4, IL6, G-CSF, and GM-CSF ↑ in mice treated with *Lactobacillus reuteri, Lactobacillus delbrueckii *and *Streptococcus thermophilus*	
APP/PS1		**♂**	6 months old mice: *Agathobacter* • *rectalis *(1 × 109 CFU/mL) was daily gavage to mice for 4 weeks		Aβ ↓ in • the *A. rectalis*- treated mice compared to PBS- treated mice	• Iba1, CD11b, and iNOS ↓ in the *A. rectalis*-treated mice compared to PBS-treated mice • Expression of the disease- associated microglia marker CD33 ↓ in the treated group	Ratio of p-Akt/Akt ↑ and ratio of p-p65/p65 • ↓ in the *A. rectalis*- treated mice compared with that in the PBS-treated mice	[101]

**↑ **incease, **↓ **decrease, **↔ **no changes

Notably, ABX treatment exclusively during P14 and P21 (without subsequent long-term ABX-treatment) was sufficient to diminish the Aβ plaque load at 6.5 months of age in the hippocampus and cortex of male APP/PS1 mice [111]. Surprisingly, these findings were not evident in 3- and 5-month-old female APP/PS1 and APP/PS1-21 mice, indicating sex-dependent effects [37, 111].

### Constant absence of the microbiota reduces Aβ depositions at the early and late stages of AD in germ-free housed mice

As an alternative to ABX-induced depletion of intestinal bacteria, GF mice can be used to analyze the effects of the absence of microbiota on AD-related pathology. In contrast to ABX-treated mice, GF mice lack any colonization with microorganisms, which allows researchers to investigate the effects of a life-long absence of microorganisms or to recolonize mice with a defined set of microorganisms at defined time points. By using GF mice, the potential side effects of ABX on innate immune cells can be avoided. However, GF-housed mice are more artificial and do not reflect a scenario that may occur outside the laboratory. However, the combination of several microbiota manipulation strategies helped to identify new mechanistic insights and improved the understanding of microbiota-dependent effects on AD.

The first publication in the context of AD research that took advantage of GF housing was published in 2017 by Harach and colleagues [55]. They reported that, compared with SPF-housed mice, GF APP/PS1 mice accumulated less Aβ in males and females [27, 55]. These results were later confirmed in 5xFAD mice housed under GF conditions (Box 1) [109]. Additionally, treatment of SPF-housed 5xFAD mice with an ABX mixture supplemented in the drinking water 2 months before the terminal endpoint of the experiment caused a reduced Aβ plaque burden compared to SPF mice at 4 months of age. However, GF 5xFAD mice displayed less Aβ pathology, in comparison to ABX-treated 5xFAD mice, indicating that the lifelong absence of microbes might have a more pronounced effect on the Aβ pathology. Additionally, GF housing and ABX treatment prevented neuronal cell loss and cognitive dysfunction during chronical disease stage (10 months of age) [109]. These findings indicate that AD can be modulated both at early stages and in a more chronic phase of the disease in a microbiota-dependent manner. Complementary, colonization of 4-month-old GF-housed APP/PS1 mice with the gut microbiota of aged WT or APP/PS1 mice increased Aβ pathology, with a more pronounced effect when the mice were colonized with microbiota derived from APP/PS1 mice [55].

In, these findings indicate that at early stages of the disease, the microbiota is involved in modulating the disease onset of AD in different mouse models via complementary microbiota manipulation strategies [55, 109–111]. Notably, this effect is dynamic and can be induced with the help of ABX. Additionally, it was revealed that GF housing and the depletion of the microbiota at late stages of the disease have beneficial effects on further disease progression in 5xFAD mice, preventing neuronal loss and memory function [27, 109].

### APP processing is not affected by the gut microbiota

One possible mechanism by which the gut microbiota may affect Aβ deposits in transgenic mouse models is by altering APP processing and subsequent Aβ production. However, no differences in APP or BACE1 expression were detected between ABX-treated and control APP/PS1 mice [110]. Further analysis revealed no changes in APP, CTF-α, CTF-β, BACE1, ADAM10 or components of γ-secretase in the hippocampal tissue extracts of 4- and 10-month-old GF, ABX-treated and SPF control 5xFAD mice [109]. These observations indicate that changes in the microbiota do not affect Aβ production in these transgenic mouse models of AD.

However, Harach and colleagues reported increased levels of the Aβ-degrading enzymes NPE and IDE in APP/PS1 GF mice compared to APP/PS1 SPF housed mice [55], which might partially explain the decreased Aβ pathology.

### Microbiota-dependent functions of microglia in mouse models of AD

After excluding APP processing as a primary mode of action, the interplay of the microbiota and CNS glial cells might impact AD pathology. Minter et al. reported that long- and short-term ABX treatment of male APP/PS1 mice reduced the number of plaque-associated microglia and astrocytes and altered microglial morphology (Table 1) [110, 111]. Additionally, modulating the interaction between the gut and the brain by housing female and male APP/PS1 mice under GF conditions reduced microglial density in the neocortex compared to colonized controls [55].

In contrast, ABX treatment of APP/PS1-21 mice did not significantly alter the number of plaque-associated microglia in 7-month-old, 3-month-old females or males [37], whereas GF 5xFAD mice showed an increase in hippocampal microglia density and plaque-associated microglia [109], indicating overall partially divergent microglial phenotypes across different AD mouse models. Furthermore, a more ramified microglial morphology was found in GF 5xFAD males than in SPF controls, indicating a rather reduced reactive state [109]. Interestingly, transcriptome analysis of cortex tissue homogenates from APP/PS1-21 mice revealed altered expression of genes associated with either homeostatic (e.g., *Mef2a*, *Junb*, *Bhlhe41*, *Fos*, and *Tnfrsf11a*) or neurodegenerative microglial phenotypes (including *Lgals3*, *C1qa*, *C1qb*, *Cd63*, and *Lag3*) in male ABX-treated APP/PS1-21 [37]. Genome-wide bulk RNA sequencing of FACS-isolated microglia from 4-month-old GF 5xFAD males revealed increased expression of genes associated with AD-related activation (*Axl*, *Cst7*, *Itgax*, *Cd9* or *Clec7a*), Aβ detection and clearance (*Apoe* and *Trem2*), and reduced expression of the homeostatic marker *P2ry12* compared with those in SPF controls [109]. Similarly, Aβ uptake was elevated in microglia from GF 5xFAD mice [41, 109], which may explain the attenuated Aβ pathology in GF-housed AD model mice.

However, the effects of the microbiota on microglia seem to be age dependent, as GF housing did not alter the (plaque-associated) microglial density or Aβ uptake in aged 5xFAD mice (10 months) compared with SPF controls [109]. The fact that ABX treatment did not affect the number of plaque-associated microglia or Aβ uptake in 5xFAD mice indicated potentially microglia-independent clearing mechanisms [109]. To expand the knowledge of these potential mechanisms, CAMs and their potential contributions to the Aβ burden were examined [141]. Although the density of these CAM populations was lower in GF and ABX-treated 5xFAD mice than in SPF 5xFAD mice, the Aβ uptake of pvMΦ was increased in GF 5xFAD mice as well as upon ABX treatment. Therefore, pvMΦ might contribute to microbiota-dependent Aβ clearance, potentially explaining the reduced Aβ pathology at disease onset as well as at a more progressed stage of the disease [109, 141]). Furthermore, pvMΦs participate in vascular Aβ uptake in a PDAPP mouse model during anti-Aβ (ED6) immunotherapy. Compared with IgG-treated controls, anti-Aβ immunotherapy promoted the recruitment of Mac387^+^ and Siglec1^+^ pvMΦs to vascular deposits and was thereby suggested to increase hemorrhagic events [165].

In addition to the Aβ clearance function of microglia, which is boosted under GF housing conditions, some studies highlight a rather harmful role of microglia in AD progression. Microglia depletion via the CSF1R inhibitor PLX5622 from 1.5 months of age reduced Aβ pathology in the cortex and thalamus of 4- and 7-monthold SPF 5xFAD mice when compared to non-treated controls, suggesting a detrimental role of microglia in controlling the Aβ burden [155]. Later, when 10-month-old 5xFAD mice were treated for 1 month with the less effective CSF1R inhibitor PLX3397, no effect on Aβ pathology was reported. However, a depletion of 80% of the microglia ameliorated dendritic spine and neuronal cell loss [156]. Additionally, in 9-week-old APP/PS1-21 males, cortical Aβ plaque load was also reduced after microglia depletion (using PLX5622) when compared to non-depleted controls [38]. In this study, the plaque-modulating effect of microglia depletion was less pronounced in 3-month-old mice, potentially indicating time-dependent effects and a detrimental role during initial plaque formation [38].

In summary, these data support, on the one hand beneficial microglial and pvMΦs functions to eliminate Aβ that are tightly controlled by gut bacteria. However, microglia and pvMΦs may detrimental upon chronic activation, whereby microglia may even contribute to Aβ propagation and spread (d´Errico et al. 2021).

### Gut-derived SCFAs modulate microglial function

Since SCFAs are known to constantly modulate microglia biology under physiological conditions [42], several studies have analyzed their impact on microglia in the context of AD. The first indication that SCFA supplementation might impact the pathogenesis of AD was published by Govindarajan et al. [51]. They injected 14-month-old APP/PS1-21 mice for 6 weeks with butyrate (1.2 g/kg body weight) and described an improved associative memory function, compared to vehicle-treated mice. Butyrate is known to inhibit histone deacetylases (HDAC) and accordingly several elevated histone acetylation (H3K14, H4K5, and H4K12) in the hippocampus and cortex of APP/PS1-21 mice compared with WT controls were detected. However, the injection of butyrate did not alter the hippocampal or cortical Aβ plaque load [51]. A more recent study showed that short-term supplementation with a mixture of acetate, propionate and butyrate enhances the Aβ plaque load in GF APP/PS1, without affecting APP processing when compared to controls [27]. This finding was confirmed in GF 5xFAD mice by supplementing acetate alone [41]. Furthermore, in both mouse models, microglia from GFhoused mice that were supplemented with SCFAs presented a more activated amoeboid morphology compared to non-supplemented GF mice [27, 41]. Importantly, in SPF housed mice, SCFA supplementation further reduced microglial Aβ uptake compared to non-supplemented SPF mice [27]. Concordantly, GF 5xFAD mice supplemented with solely acetate showed a diminished Aβ uptake comparable to the levels found in SPF mice [41]. Thereby, acetate was identified as the microbiota-derived SCFA modulating microglia Aβ uptake [41].

Additionally, in APP/PS1, an altered expression of genes associated with pathogen recognition, such as *Tlr7*, *Tyrobp* and *Cd14*, and downregulation of *Myd88,* was observed. Furthermore, they showed an upregulation of activation (*CD86*) and phagocytosis markers (*CD68*), as well as an activation of the AD relevant APOE-TREM2 pathway [27].

However, Colombo et al. and Erny et al. reported different effects on microglial density in different mouse models. In GF APP/PS1 mice, short-term SCFA supplementation resulted in an increase of plaque-associated microglia [27], whereas acetate supplementation decreased the number of microglia in a GF 5xFAD mouse model [41]. Interestingly, Erny and colleagues reported equal (plaque-associated) microglia density and Aβ pathology in SPF 5xFAD mice and GF 5xFAD mice supplemented with acetate, indicating a regulatory effect of acetate [41]. Furthermore, in acetatetreated GF 5xFAD mice microglia exhibited an increased cytokine expression and reduced phagocytic capacity when compared to non-treated GF housed mice.

Under physiological conditions, the absence of the microbiota with subsequently reduced acetate levels in the brain alters the metabolic state of microglia toward an abnormal amino acid metabolism with impaired arginine, proline and purine metabolism. Notably, H3K4me3 and, more prominently, H3K9ac in microglia were affected by the gut microbiota, including metabolic genes, indicating that the metabolic fitness of microglia is at least partially epigenetically controlled (Fig. 1). Furthermore, metabolite profiling of FACS-isolated microglia revealed a decrease in various fatty acids and lipids in GF mice compared with SPF mice. Moreover, microglia from GF mice have an increased number of mitochondria with a reduced mitochondrial membrane potential and an increased production of mitochondrial ROS compared with SPF microglia [41]. These mitochondrial defects can be ameliorated by acetate supplementation, which fuels the mitochondrial TCA cycle and reduces GF-associated impairments in complex II of the respiratory chain. Interestingly, complex II defects could not be detected under pathological conditions in the 5xFAD mouse model, suggesting an altered metabolic state during disease conditions.

In addition to studies performed in mice, SCFAs have various crucial functions across species, as highlighted in a study that housed pigs under GF conditions. The absence of a microbiota and the subsequent lack of SCFAs impaired physiological functions in the gut, including colonic mobility, blood flow and gastrointestinal pH, which affected the absorption of electrolytes and nutrients. The transplantation of a healthy microbiota rescued the nutrient digestibility and improved the intestinal development and barrier function [193].

### Microbiota-derived tryptophan metabolites ameliorate AD pathology

It has been suggested that tryptophan-derived derivatives such as indole-3-lactic acid, indole-3-acetic acid and indole-3-carbinol can affect AD pathology [125]**.** Recently, tryptophan and indole-3-lactic acid (ILA) were identified as critical gut-derived molecules that probably reduce Aβ accumulation and cognitive impairment via AhR signaling [82]. Additionally, the oral administration of *Streptococcus thermophilus, Lactobacillus reuteri*, and *Lactobacillus delbrueckii* to 5xFAD mice could reduce soluble Aβ42 levels in aged animals. This administration correlated with increased levels of tryptophan and ILA in the plasma. Furthermore, a clinical data set analyzing feces, showed that *Lactobacillus reuteri* is more abundant in healthy individuals than in patients with an early stage of Aβ accumulation. Moreover, postmortem brain analyses revealed increased expression of genes associated with the AhR signaling pathway, suggesting a potential protective effect against AD progression, recapitulating the murine findings [82].

### Microbiota-dependent functions of astrocytes in AD mouse models

**Fig. 2:**
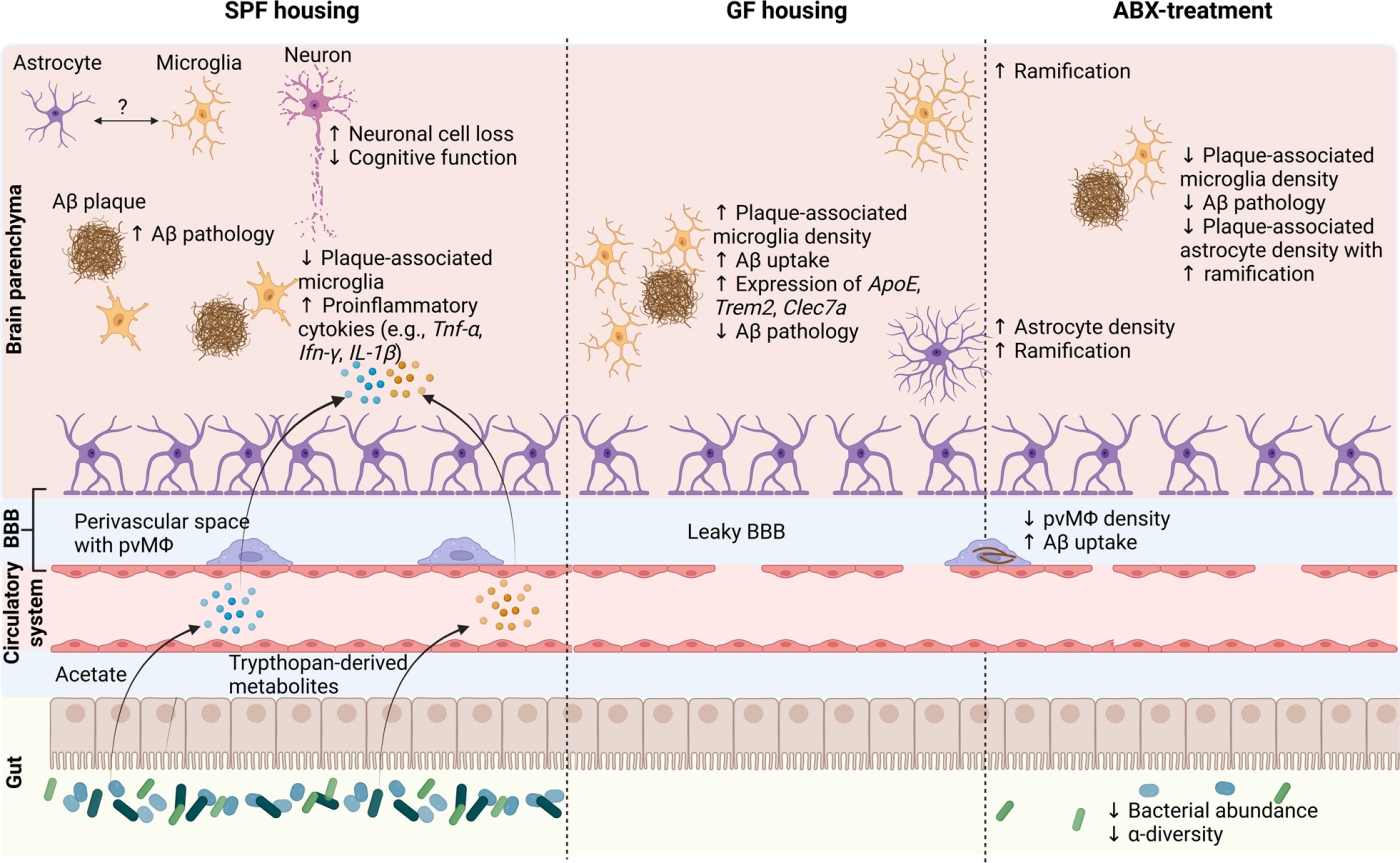
Gut-microbiota driven alteration in the development of AD. The absence of gut microbiota via germ-free (GF) housing or its reduction via antibiotic-treatment (ABX), results in a reduction of gut microbiota produced short-chain fatty acids (SCFAs) and tryptophan-derived metabolites. Microglial Aβ clearance is controlled by gut-derived acetate. Additionally, the cell density of perivascular macrophages (pvMΦ) is reduced, accompanied with an increased Aβ uptake upon GF-housing and ABX-treatment, resulting in ameliorated AD pathology

In addition to microglia, astrocytes are modulated by the gut microbiota in the APP/PS1 mouse model. Short-term ABX treatment altered the number of plaque-associated astrocytes and their morphology **(**Fig. 2**)** [110]. Recently, Chandra et al. performed a more in-depth analysis of the effects of short-term ABX treatment and GF housing on astrocytes in the APP/PS1-21 mouse model [24]. Compared with vehicle control-treated male APP/PS1-21 mice, 9-week- and 3-month-old short-term ABX-treated male APP/PS1-21 mice presented a reduced number of plaque-associated astrocytes in the cortex. Fecal microbiota transplantation (FMT) from non-ABX-treated APP/PS1-21 mice into short-term ABX-treated APP/PS1-21 mice restored the ABX-induced changes in astrocyte cell numbers. No differences were observed in females, indicating sex-specific effects. Compared with those in mice raised under SPF conditions, plaque-associated astrocytes were decreased in 9-week-old APP/PS1-21 males housed with GF, whereas no differences were detected in the number of nonplaque-associated astrocytes [24]. Additionally, ABX treatment and GF housing similarly increased the morphology of astrocytes compared with vehicle-treated and SPF-housed APP/PS1-21 mice. Interestingly, in short-term ABX-treated microglia-depleted (PLX5622) 3-month-old APP/PS1-21 mice, no astrocytic morphological alterations were observed compared with those in vehicle-treated mice. However, the previously described reduction in plaque-associated astrocytes was maintained, indicating the existence of microglia-independent and microglia-dependent mechanisms [24]. In general, the gut microbiota-dependent interactions of astrocytes and microglia are not yet fully understood in the context of AD.

Several studies have focused on the impact of SCFAs on microglia; however, astrocytes can also be modulated by SCFAs. Sun and colleagues first demonstrated this effect by performing long-term dietary SCFA supplementation in APP/PS1 mice [159]. They reported a significant improvement in learning and spatial memory abilities, as well as a reduction in Aβ pathology. Additionally, the SCFA diet upregulated astrocytic glutamine synthetase, indicating increased metabolic astrocyte–neuron coupling [159].

### Microbiota-derived effects on tau pathology

Previous studies focused mainly on Aβ pathology, whereas a recent study determined the effect of the gut microbiota on tau pathology in a P301S tau transgenic mouse model with humanized ApoE (Box 1) [146]. Human *APOE* is expressed in three major genetic isoforms, whereby *APOE4* is considered to be the most important genetic risk factor for AD [137]. Short-term ABX treatment and GF housing ameliorated tau pathology in 40-week-old P301S tau mice, resulting in improved behavior and inhibited neuronal cell loss. ABX treatment showed sex differences, with reduced levels of phosphorylated hippocampal tau found only in males. Furthermore, and in accordance with previous studies, SCFA supplementation in GF P301 tau mice with the *APOE4* isoform exacerbated AD pathology [146]. Overall, this study hypothesized that the gut microbiota can regulate the immune response to tau pathology potentially via SCFAs, but the underlying mechanisms remain to be determined.

### Microbiota dysbiosis and its impact on cognitive function

Several studies have shown that the microbiota composition of AD mice is altered compared to WT mice. To address the question if the transmission of the AD-associated microbiota is sufficient to impact cognitive function in WT animals, Zhang and colleagues performed cohousing studies [191]. Specifically, they cohoused two-month-old WT female mice with sex- and age-matched 5xFAD mice for a period of 3 months, caused a cognitive impairment of the WT animals. Furthermore, an increased abundance of *Dubosiella* and *Peptococcaceae* was reported. These bacteria are known to be associated with inflammation, decreased amounts of butyrate, increased levels of tau phosphorylation and increased accumulation of Aβ42. Additionally, *Bacteroides fragilis* was reported to trigger an AD-like pathology with cognitive dysfunction in mice [181]. However, there are also contradictory studies suggesting a decrease in *Bacteroides* species [97, 194]. Additionally, new studies have shown that *Agathobacter*, a butyric acid-producing bacteria belonging to the *Lachnospiraceae* family, might have a neuroprotective effect. In APP/PS1 mice, the administration of *A. rectalis* reduced Aβ accumulation by inhibiting microglial activation [101]. The authors suggested that the underlying mechanism could be the butyrate-dependent regulation of the Akt/NF-κB pathway.

A critical role for the microbiota was further determined in zebrafish, whereby zebrafish housed under GF conditions failed to develop physiological social behavior due to impaired microglial remodeling [18]. Interestingly, microglial density was lower in GF zebrafish larvae compared to colonized controls, which contrasts with findings in mice [18, 42]. Furthermore, monocolonization with distinct bacterial strains (e.g., *Aeromonas veronii*, *Enterobacter cloacae*, and *Staphylococcus sp*.) could restore neurodevelopmental features, indicating a dynamic bacterial effect on microglia and CNS development [18].

### Microbiota-dependent alteration of the peripheral immune system

Data from clinical and preclinical studies indicate that the peripheral immune system might be involved in AD. Epidemiological studies revealed a dysregulated peripheral immune system in AD patients [86], with various inflammatory markers, peaking during the initial symptomatic phase [149]. Additionally, the risk to develop AD is increased by the dysregulation of peripheral immune markers, including C-reactive protein, IL-6 and IL-1β [31, 149]. Furthermore, patients with a history of infectious disease necessitating hospitalization have an increased AD risk [153]. In addition to epidemiological studies, animal studies underline the interplay of the peripheral immune system and AD. Generally, it is known that peripheral bacterial and viral infections can induce inflammatory pathways in the brain [8, 74]. In APP/PS1 mice, a systemic inflammatory stimulus via LPS activated microglia and impaired their Aβ clearance capacity [166]. In contrast, sequential low-dose LPS stimulation can induce immune memory in microglia cells, causing a tolerance effect [178]. Furthermore, repetitive low-dose LPS stimulation reduced Aβ pathology and neuronal cell death in the APP23 AD mouse model [178]. These effects are reportedly mediated via epigenetic alterations of microglia. Increased acetylation of H3K27 and methylation of H3K4, indicating active enhancers, were altered and associated with endocytosis and phagocytosis. In contrast, a single LPS stimulation worsens Aβ pathology and alters the epigenetic landscape of microglia toward the upregulation of pathways associated with TNFα and HIF-1 signaling.

To investigate the impact of the microbiota on the peripheral immune system and subsequently on the pathogenesis of AD, further studies were performed in APP/PS1 and APP/PS1-21 mice. In both mouse models, short-term and long-term ABX-treatment altered the serum concentrations of various cytokines and chemokines in a sex-specific manner [37, 110, 111]. However, these studies reported partially contrary results, and these findings need to be clarified in future studies.

### Alterations of the human gut microbiota in AD patients

In addition to data obtained from rodents, studies with human AD patients have shown a potential interplay between the gut microbiota and AD (Table 2). Initially, two studies published in 2017 revealed alterations in the gut microbiota composition in AD patients. First, Cattaneo et al. reported an increased abundance of *Escherichia* and *Shigella* species and decreased levels of *Eubacterium rectale* compared with non-AD controls [22]. Second, Vogt et al. performed 16S rRNA sequencing of fecal samples and reported decreased microbiota diversity in AD patients. Furthermore, they described an increase in *Bacteroidetes* and a decrease in *Firmicutes*. [173]. Reduced abundance of *Firmicutes* was also found in another Chinese study including patients with mild cognitive impairment (MCI) and AD [98]. However, other studies reported a decrease in *Bacteroidetes* [97, 194]. It was further suggested that the gut microbiota composition might change already early in the disease process and correlated rather with Aβ and tau levels, and to a lesser extent with the degree of neurodegeneration [44].

**Table 2:** Studies of the gut microbiota composition in AD patients

Publication	Main Findings		HC			AD				Location
			N	Sex (M/F)	Age	Stage	N	Sex (M/F)	Age	
Cataneo et al., 2017	Aβ^+^ MCI vs. Aβ^-^ MCI and HC: ↓ *Eubacterium rectale* ↑ *Esherichia*/*Shigella*		10	4/6	68 ± 8	Aβ^-^ MCI	34	16/18	70 ± 7	Lombardy, Italy
						Aβ^+^ MCI	40	20/20	71 ± 7	
[173]	AD parents vs. HC: ↓ *Firmicutes *and *Bfidobacterium* ↑ *Bacteroidetes*		25	7/18	69.3 ± 7.5	AD	25	8/17	71.3 ± 7.3	Wisconsin, USA
[194]	AD parents vs. HC: ↓ *Bacteroidetes* ↑ *Actinobacteria*		43	23/20	69.7 ± 9.2	AD	43	23/20	70.1 ± 8.8	Chongqing, China
[98]	AD parents vs. HC: ↓ *Firmicutes* ↑ *Proteobacteria*		32	16/16	76.9 ± 9.4	MCI	32	14/18	70.5 ± 11.0	Hangzhou, China
						AD	33	19/14	74.9 ± 11.4	
[59]	AD parents vs. HC: ↓ *Butyrivibrio*, *Eubacterium *and *Clostridium* ↑ *O. splanchnicus*, *K.pneumoniae*, *B.fragilis *and *E. lenta*		51	8/43	80.3 ± 10.2	AD	24	4/20	84.7 ± 8.1	Massachusets, USA
[97]	AD and MCI parents vs. HC: ↓ *Bacteroidetes* ↑ *Esherichia, Bfidobacterium *and *Lactobacillus*		30	13/17	63.9 ± 5.1	MCI	30	12/18	65.4 ± 7.6	China
						AD	30	15/15	66.3 ± 5.1	
[66]	AD parents vs. HC: ↓ *Enterococcaceae *(genera: *Megamonas*, *Enterococcus *and *Anaerostipes*) ↑ *Proteobacteria *(orders: *Enterobacteriales*, *Deltaproteobacteria *and *Desulfovibrionales*; families: *Enterobacteriaceae *and *Desulfovibrionaceae*; genera: Escherichia/*Shigella*, *Ruminococcaceae_UCG_002*,		47	22/25	71.7 ± 6.7	AD	30	17/13	71.9 ± 6.9	Chengdu, China
	*Shuttleworthia*, *Anaerofustis*, *Morganelia*, *Finegoldia*, and *Anaerotruncus*)									
[193]	AD parents vs. HC: ↓ *Odoribacter*, *Anaerobacterium *and *Papillibacter* ↑ *Bifidobacterium*, *Sphingomonas*, *Lactobacillus *and *Blautia*		32	14/18	71.1 ± 5.9	AD	60	24/36	72.8 ± 7.3	Beijing, China
[73]	Aβ^+^ cognitive normal vs. Aβ^-^ cognitive normal ↓ *Victivallis*, *Enterococcus*, *Mitsuokella *and *Erysipelotrichaceae* ↑ *Megamonas*, *Serratia*, *Leptotrichia *and *Clostridiaceae*		60	31/29	72.9 ± 6.8	Aβ^+^ cognitive normal	18	7/11	75.2 ± 7.1	Republic of Korea
[44]	Preclinical AD vs. healthy individuals ↑ *Dorea formicigenerans, Oscillibacter *sp. 57_20, *Faecalibacterium prausnitzii, Coprococcus catus, Anaerostipes hadrus* ↓ *Methanosphaera stadtmanae*		115	49/66	77.02 ± 5.80	Aβ^+^ cognitive normal	49	24/25	78.96 ± 4.51	St-Louis (MO) USA

In summary, these correlative studies describe alterations of the microbiota composition of AD patients. However, the alterations seem to be partially inconsistent across studies, and it remains unclear if and to what extent the reported dysbiosis affects AD progression including bacteria-derived molecules that may affect the AD pathology. In addition to AD, several other CNS diseases have been shown to be affected by the gut microbiota.

### Parkinson’s disease and the role of the gut microbiota

In addition to AD, other neurodegenerative diseases, including Parkinson’s disease (PD), are influenced by the microbiota. Among other symptoms, PD patients develop bradykinesia, rigidity, postural instability, and tremors in the hands, arms and legs. Pathologically, PD is characterized by a loss of dopaminergic neurons in the substantia nigra and an accumulation of intracellular protein deposits (Lewy bodies). Interestingly, in PD patients, constipation is among the first symptoms, partially preceding neurological symptoms by decades [2]. Furthermore, the composition of the gut microbiota is altered in PD patients, with an increase in *Enterobacteriaceae*, *Lactobacillus*, *Bifdobacterium*, and *Akkermansia* [117, 148, 169]. Additionally, opportunistic pathogens, including *Corynebacterium*, *Porphyromonas*, *Alistipes*, *Bacteroides*, *Escherichia*, *Megasphaera* and *Desulfovibrio*, are elevated in PD patients. Moreover, various bacteria, e.g., *Blautia*, *Coprococcus*, *Roseburia*, *Lachnospira*, and the SCFA-producing *Fusicatenibacter* and *Faecalibacterium, are decreased* [117, 148, 169]. Consistent with this reduction, decreased fecal SCFA levels can be found in PD patients. Interestingly, the reduction of SCFA-producing bacteria in PD patients is correlated with the severity of cognitive and motor symptoms [26, 170]. However, SCFA levels in the plasma of PD patients are increased, potentially because of elevated intestinal permeability. In addition, PD patients presented increased expression of several inflammatory markers in the colon and feces, including CCL2, CCL5, CCR5, IL-1β, IL-6, IL-8, IL-17A, IFN-β, IFN-γ, TNFα, TLR2, and TLR4, as well as increased numbers of CD3^+^ T cells in the colon [65, 126, 145]. Furthermore, in rodent and nonhuman primate studies, the intraintestinal injection of various forms of misfolded alpha-synuclein can reach the locus coeruleus and substantia nigra via the vagus nerve [60]. This transmission can be mitigated by surgical vagotomy [83]. Moreover, a cohort of patients in Denmark who underwent vagotomy from 1977–1995 presented a decreased risk for PD [160].

In a mouse model overexpressing α-syn (Thy1-aSyn), ABX treatment (from 5–6 weeks of age until 12–13 weeks) ameliorated α-syn-dependent motor deficits and reduced microglia activation compared to non-treated mice [140]. In the same study it was shown that GF housing reduced motor deficits, α-syn accumulation and microglia activation in comparison to SPF mice, effects which could be reversed via SCFA supplementation. Additionally, transplantation of faces obtained from PD donor patients worsened motor deficits more prominently in Thy1-aSyn mice than in mice transplanted with faces from matched healthy donors [140]. FMT from toxin-induced PD mice (MPTP) into WT mice can also impair their motor function [158].

### Microbiota-dependent effects on multiple sclerosis

Multiple sclerosis (MS) is considered an autoimmune disease of the CNS whose pathogenesis is also influenced by the microbiota. MS is characterized by demyelination and inflammation of the CNS and affects approximately 2.8 million people worldwide [174]. Various studies have reported alterations in the microbiota of MS patients, with increases in *Akkermansia*, *Methanobrevibacter*, *Ruthenibacterium lactatiformans*, *Hungatella hathewayi*, and *Eisenbergiella tayi* [11, 68] and decreases in SCFA-producing *Butyricimonas*, *Faecalibacterium*, and *Clostridium* cluster IV and XIVa [68, 112]. Similarly, fecal SCFA levels are slightly lower in MS patients than in controls [68]. In the serum of MS patients, butyrate levels are dampened [142], however, acetate levels are increased [127]. These results indicate that alterations in the gut microbiota are associated with MS risk and disease progression. However, a robust conserved pattern of microbiota alterations remains to be defined.

Additionally, in a monophasic experimental autoimmune encephalomyelitis (EAE) mouse model, the gut microbiota affects MS pathogenesis. ABX treatment [185] and the absence of complete microbiota caused by GF- housing attenuated EAE and spontaneous MS pathogenesis while reducing the expression of proinflammatory cytokines [12, 94]. Mechanistically, these beneficial effects are caused by a reduced T and B-cell response as well as a more pronounced T reg response [12, 94]. Interestingly, SCFA supplementation (especially propionate) increased the Treg population, decreased Th1 and Th17 cells, and ameliorated the annual relapse rate and brain atrophy in human MS patients [39].

In a cuprizone toxin-induced demyelination model, GF housing reduced microglial cell numbers and increased oligodendrocyte density, an effect that was reversible upon colonization [107]. Additionally, the colonization of GF-housed EAE mice with MS patient faces worsened symptoms more intensely than FMT from healthy individuals did [23].

In addition to SCFAs, AhR ligands were identified as further microbiota-derived metabolites (produced by, e.g., *Peptostreptococcus russellii* and *Lactobacillus spp*. [3]) that modulate the pathogenesis of EAE [133]. AhR signaling increases TGF-α and VEGF-B production in microglia, eliciting proinflammatory neurotoxic astrocyte activities and thereby subsequently worsening EAE [133, 134]. In addition to the impact of the gut microbiota on EAE, the microbiota residing in the lung influences the outcome in a rat model of EAE. Lung dysbiosis was induced by intratracheal neomycin treatment and subsequently caused decreased infiltration of B cells and CD4^+^ and CD8^+^ T cells into the CNS. Furthermore, microglial type I IFN activation is dampened, hence ameliorating the severity of EAE [64].

### Outlook: from mouse models to potential AD treatment strategies

Modulating microglia and CAMs directly or indirectly might be a promising strategy to treat AD and other CNS diseases. The gut microbiota is able to impact microglia; therefore, altering the gut microbiota composition or providing directly beneficial bacteria-derived molecules might be a promising strategy to adjust microglia for the treatment of CNS diseases. The strategies used to alter the gut microbiota might include probiotics/prebiotics, FMT, diet or the direct application of beneficial bacterial molecules (Duscha et al. 2020).

In APP^NL−G−F^ mice, oral supplementation with *Bifidobacterium breve* (for 4 months beginning at 3 months of age) reduces microglial cell density in the hippocampus and ameliorates the hippocampal Aβ plaque load and memory impairment [1]. The beneficial effects of probiotics were further shown in (i) APP/PS1 mice (supplementation with *Bifidobacterium Lactis Probio-M8* for 45 days starting at 4 months of age decreased the cortical Aβ plaque load and improved cognitive performance, as determined via the Y-maze test) [20] and (ii) 3xTG AD mice (supplementation with SLAB51, a mixture of 9 bacterial strains, for 4 months starting at 8 weeks of age decreased the Aβ plaque load and improved cognitive performance, as determined via novel object recognition) [13].

Furthermore, modulating the gut microbiota composition via the drug sodium oligomannate (GV-971) was reported to ameliorate AD pathogenesis in 5xFAD mice. Wang et al. gavaged 6-month-old 5xFAD mice for one month with GV-971 (Table 1) and reported decreased hippocampal Aβ and tau accumulation, improved cognitive performance, and decreased microglial density in the hippocampus when compared to untreated 5xFAD mice [176]. A recent publication confirmed the beneficial effects of sodium oligomannate treatment on AD pathogenesis in APP/PS1 mice. Daily treatment with sodium oligomannate starting at the age of 8 weeks reduced cortical Aβ pathology in 12-week-old males and females. Although the beneficial effect on Aβ accumulation was less pronounced in females [14].

In addition to probiotics and prebiotics, diet is an important influencing factor on the microbiota composition and has been shown to influence the pathogenesis of AD in various mouse models. A high-fat diet was reported to exacerbate the Aβ plaque load in APP/PS1 [17], 5xFAD [108] and APP^NL−F^ mice [105]. In contrast, a ketogenic diet can decrease the Aβ plaque load and ameliorate cognitive performance and neuronal cell loss in 5xFAD mice [182]. A ketogenic diet is characterized by nearly complete elimination of carbohydrates, which results in ketone body production and their consumption as main energy source. Among various ketone body species, β-hydroxybutyrate is the most abundant. β-Hydroxybutyrate is known to inhibit the NLRP3 inflammasome [188], which is chronically activated in various AD mouse models [59]. Compared with vehicle treatment, β-hydroxybutyrate supplementation via drinking water (8 weeks, 0.01875 g/ml) not only reduced inflammasome activation in 5xFAD mice but also decreased the Aβ plaque load. [150]. Furthermore, β-hydroxybutyrate supplementation decreases the density of (plaque-associated) microglia in 5xFAD mice. Interestingly, β-hydroxybutyrate supplementation had no effect on the microglia of WT mice [150].

In addition to dietary interventions, intermittent fasting can modulate the gut microbiota and, e.g., increase the amount of *Firmicutes* while decreasing B*acteroidetes* when compared to mice fed ad libidum. Furthermore, intermittent fasting reduces microglial density and subsequently ameliorates Aβ pathology and cognitive dysfunction in 5xFAD mice. Furthermore, supplementation with amino acids found to be increased during intermittent fasting (sarcosine and dimethylglycine) can mimic the beneficial effects of intermittent fasting [123].

Treatment strategies might include the modulation of microglia, potentially by altering the microbiota via prebiotics, probiotics or diet intervention. Furthermore, the phagocytic activity of microglia can be increased in aging mice via CD22-blocking antibodies. Thereby, the uptake of myelin debris, amyloid-β oligomers and α-synuclein fibrils could be increased, subsequently improving cognitive function [128]. A further strategy is based on the depletion of dysfunctional microglia, followed by the engraftment of hematopoietic cells. A recent study reported exacerbated Aβ pathology and reduced microglia clustering around plaques in TREM2-deficient 5xFAD mice. They then depleted the microglia in TREM2-deficient 5xFAD mice via CSF1R inhibition and replaced them by infusing TREM2^WT^ hematopoietic cells. After engraftment, these circulation-derived myeloid cells clustered around plaques and reduced the Aβ pathology when compared to mice transfused with TREM2^KO^ hematopoietic cells [186].

In summary, the abovementioned studies pave the way for an improved understanding of how microglia and their interactions with the microbiota can support the development of future AD, PD and MS treatments.

## Data Availability

No datasets were generated or analysed during the current study.
